# An Experimental Decision-Making Paradigm to Distinguish Guilt and Regret and Their Self-Regulating Function via Loss Averse Choice Behavior

**DOI:** 10.3389/fpsyg.2012.00431

**Published:** 2012-10-22

**Authors:** Ullrich Wagner, Lisa Handke, Denise Dörfel, Henrik Walter

**Affiliations:** ^1^Division of Mind and Brain Research, Department of Psychiatry and Psychotherapy, Charité – Universitätsmedizin BerlinBerlin, Germany

**Keywords:** guilt, regret, social decision-making, game theory, loss aversion, emotion regulation

## Abstract

Both guilt and regret typically result from counterfactual evaluations of personal choices that caused a negative outcome and are thought to regulate human decisions by people’s motivation to avoid these emotions. Despite these similarities, studies asking people to describe typical situations of guilt and regret identified the social dimension as a fundamental distinguishing factor, showing that guilt but not regret specifically occurs for choices in interpersonal (social) contexts. However, an experimental paradigm to investigate this distinction systematically by inducing emotions of guilt and regret online is still missing. Here, extending existing procedures, we introduce such a paradigm, in which participants choose in each trial between two lotteries, with the outcome of the chosen lottery (gain or loss) being either assigned to themselves (intrapersonal trials) or to another person (interpersonal trials). After results of both the chosen and the unchosen lottery were shown, subjects rated how they felt about the outcome, including ratings of guilt and regret. Trait Guilt (TG) was determined for all participants in order to take their general inclination to experience guilt into account. Results confirmed that guilt but not regret specifically occurred in an interpersonal context. Percentages of loss averse choices (choosing the lottery with the lower possible monetary loss) were determined as indicators of regulation via guilt and regret avoidance. High TG scorers generally made more loss averse choices than low TG scorers, while trial-by-trial analyses showed that low TG scorers used their feelings of guilt more specifically to avoid the same emotional experience in subsequent choices. Our results confirm the social dimension as the critical factor distinguishing guilt from regret and identify TG as an important moderator determining the way in which guilt vs. regret can regulate their own occurrence by influencing choice strategies.

## Introduction

Guilt and regret are two closely related emotions. In everyday life, they tend to co-occur, which may be the reason why many people would find it difficult to distinguish between them conceptually. In their analysis of regret, Gilovic and Medvec (1995) expressed this relatedness by proposing that feelings of regret are “likely to be tinged with guilt.” In fact, guilt and regret share essential features. One commonality is that both emotions typically occur in situations when one feels responsible for a negative outcome or harm, which could have been avoided if one had chosen a different action. Thus, both guilt and regret are based on counterfactual choice evaluation, i.e., a comparison of an actual outcome of a choice with what could have happened in case of an alternative choice. Not surprisingly, both emotions accordingly also share essential phenomenological characteristics. For example, in a study by Russell and Mehrabian (1977) participants were asked to rate various emotions on a semantic differential (including pleasure–displeasure, arousal–non-arousal, and dominance–submissiveness). No difference between guilt and regret was found for any of the emotion features. Both guilt and regret, in contrast to most other emotions, are also closely related to agency and personally experienced responsibility. For example, Frijda et al. (1989), comparing appraisal components of 32 emotions, found that guilt and regret differed from all other emotions (including shame) by a particularly strongly associated experience of self-agency. Notably, guilt and regret also share an important functional feature: experiencing them is thought to regulate subsequent behavior by a motivation to avoid the occurrence of these feelings after future choices (Zeelenberg et al., 1996; Coricelli et al., 2005; Ellingsen et al., 2010; Chang et al., 2011). Of course, people are generally motivated to avoid negative emotional states, but because guilt and regret are so closely linked to personal choice behavior and therefore also to personal responsibility and control (which is much less the case for other typical negative emotions like anger or fear), people can actively and deliberately adopt appropriate choice strategies to regulate (i.e., reduce) the probability of experiencing them in the future. From this perspective, both guilt and regret can be regarded as “self-regulating emotions.”

Nevertheless, despite all these similarities, two studies performing a closer analysis of the psychological determinants of guilt and regret revealed one critical dimension that clearly distinguishes between guilt and regret, namely the social dimension (Berndsen et al., 2004; Zeelenberg and Breugelmans, 2008). In this context, social dimension refers to the target of the negative outcome or harm that one caused, i.e., whether the negative outcome affected oneself (intrapersonal or non-social condition) or another person (interpersonal or social condition). Both of the studies showed that when this factor is taken into account, guilt can be clearly distinguished from regret in being strongly associated with interpersonal but not intrapersonal harm, while no such specificity is found for regret. In one of the experiments, Zeelenberg and Breugelmans, 2008, Study 1) presented their subjects with two versions of a hypothetical scenario, asking them how they would feel in the respective situation. Specifically, participants were asked to imagine that they had left their clothes and shoes in the bathroom after taking a shower, and that later they themselves (intrapersonal condition) or their mother (interpersonal condition) would stumble over them, leading to a broken foot and unbearable pain. There was a highly significant interaction between the person suffering from harm (self vs. mother) and the emotion (guilt vs. regret). In particular, subjects rated substantially higher guilt but not regret for the interpersonal as compared to the intrapersonal condition. Similar patterns were found for emotion ratings of actual events that subjects recalled from their own past. Some data in the studies by Berndsen et al. (2004) pointed to an opposite pattern for regret than for guilt, i.e., stronger ratings after intrapersonal than after interpersonal harm, but this could not be confirmed by Zeelenberg and Breugelmans’ (2008) data. Together, these results are in line with Baumeister et al.’s (1994) analysis of guilt as an inherently interpersonal emotion, which occurs in social relationships and helps to maintain them, although the specific difference between guilt and regret was not directly addressed in this analysis.

Based on the distinction between intra- and interindividual harm, the primary aim of the present study was to develop an experimental paradigm that allowed us to differentially induce guilt and regret online in a systematic manner as a result of subjects’ actual choices during the experiment. Generally, it is quite a challenge to induce these emotions in standardized laboratory settings, especially for guilt, because this requires subjects to act in a way that makes them feel responsible for a damage to another person, which they would naturally avoid (particularly with the knowledge that one’s behavior is continuously registered by an experimenter). To circumvent these problems, several procedures have been developed to induce feelings of guilt without directly linking them to choices made online within an experimental paradigm. Such procedures encompass the imagination of hypothetical scenarios (Takahashi et al., 2004; Moll et al., 2007; Kedia et al., 2008), giving false feedback (Amodio et al., 2007), reading newspaper articles (Stillman and Baumeister, 2010), or autobiographical memory paradigms, in which subjects write down specific emotional events from their own past (De Hooge et al., 2007; Gangemi et al., 2007; Nelissen et al., 2007) or are directly asked to specifically relive the emotions from such personal past events (Shin et al., 2000; Wagner et al., 2011). Especially the latter method is well suited to induce relatively intense feelings of guilt in the laboratory, because the strongest guilt-inducing stimuli are selected individually and refer to events that actually happened rather than merely being hypothetical situations (Wagner et al., 2011). In the present study, however, we were specifically interested in the occurrence of guilt and regret in the context of actual choices within the experimental setting. In this way, we would induce these emotions in a manner more fitting to simulate their natural occurrence, which is typically linked to individual choices. Furthermore we would also be able to analyze the consequences of experiencing guilt and regret on the regulation of subsequent choice behavior in repeated conditions.

For this purpose, we used a decision-making paradigm in which subjects repeatedly make choices with real monetary effects. Such paradigms were originally developed within the framework of game theory (von Neumann and Morgenstern, 1944) and are meanwhile frequently used in the fields of social neuroscience, neuroeconomics, and decision-making research to model the dynamics of choice behavior as well as to analyze the underlying neural mechanisms (Fehr and Camerer, 2007; Sanfey, 2007). In recent economic research, emotional factors like guilt and regret are sometimes incorporated into such models as parameters in mathematical formulas of utility functions developed to optimize the prediction of choice behavior in certain game-theoretical paradigms (Coricelli et al., 2005; Krajbich et al., 2009; Chang et al., 2011). However, even though these studies demonstrate that the role of guilt and regret in economic choice behavior has basically been acknowledged, participants in this type of research are typically not directly asked for their specific emotions after they have made a choice and got feedback about the outcome. Here, complementing these neuroeconomic approaches, we obtained participants’ ratings regarding their feelings of guilt and regret, which allowed us to directly test the assumed interpersonal specificity of subjective guilt experiences in actual decisions. Although guilt and regret have already been addressed separately in a variety of studies of choice behavior, there is currently no decision-making paradigm that directly compares them as possible factors in their effects on choice behavior.

To develop such a paradigm, we relied on a well-established procedure from Coricelli and coworkers (Camille et al., 2004; Coricelli et al., 2005) that has been used to investigate (intrapersonal) regret, extending it by an interpersonal (social) condition to induce guilt. Within each trial of this paradigm, subjects choose which of two lotteries is to be played. For each of the two lotteries, the amount of money that can be won or lost is indicated on the screen, as well as the respective probabilities of winning or losing. After the decision, the selected lottery is played and the outcome (won or lost amount of money) is added/subtracted from the overall earnings of the subject. Apart from the outcome of the actually played lottery, the outcome of the non-selected lottery is also shown. This procedure, as applied in the original version of the paradigm developed by Coricelli et al. (2005), is well suited to induce regret, which is expected to occur when the outcome of the non-selected lottery would have been better than that of the selected lottery due to counterfactual evaluation. However, all outcomes are only attributed to the participant himself/herself in this original paradigm, so this would constitute only intrapersonal regret, as the possible negative outcome does not affect anybody else. We therefore introduced an interpersonal (social) condition, in which the decision was not made for oneself but for another person. In order to maximize the probability and extent of feelings of guilt in this interpersonal condition, we designated as the other person a young child, Anastasia, in need of expensive medical treatment for which a local organization was collecting donations (see Materials and Methods, for details). In each trial, the outcome was either assigned to the subject (“self” condition) or to Anastasia (“other” condition), so that gains and losses were independently determined for the two conditions. The assignment was always announced at the beginning of each trial, before the subject made his or her choice. In control conditions (no responsibility), the computer made a random choice, and the subject just watched what happened on the screen. In the end of each trial, subjects were asked to rate how they felt about the outcome with respect to different emotions, including ratings of guilt and regret.

In short, our paradigm basically used the well-known Coricelli procedure, but extended it in two ways in order to allow a distinction between guilt and regret according to previous psychological research (Berndsen et al., 2004; Zeelenberg and Breugelmans, 2008). First, an interpersonal (social) condition was introduced in addition to the intrapersonal (non-social) condition. Second, specific emotion ratings were obtained after each trial, allowing us to directly test the psychological patterns of guilt vs. regret experiences from the studies of hypothetical scenarios and descriptions of personally recalled events within a behavioral decision-making paradigm. According to the results from Berndsen et al. (2004) and Zeelenberg and Breugelmans (2008), we hypothesized that guilt, but not regret, would be substantially more pronounced in the interpersonal than in the intrapersonal condition after negative outcomes^1^. In order to take individual *a priori* differences in the inclination to experience guilt into account, we also assessed Trait Guilt (TG) in each subject (Kugler and Jones, 1992; Jones et al., 2000). We expected stronger guilt feelings in individuals with high TG scores than in individuals with low TG scores after own choices with negative outcomes.

A second aim in this study was to analyze regulating effects of guilt and regret on subsequent choice behavior. As mentioned, both guilt and regret are thought to affect subsequent behavior by a motivation to avoid their occurrence in future choices (Zeelenberg et al., 1996; Coricelli et al., 2005; Ellingsen et al., 2010; Chang et al., 2011) and thus have the capacity to (down-)regulate themselves on the long run. In the present paradigm, there were only very limited options to act, and we held the expected values of the two lotteries within a trial constant, so there were also not many possible criteria on which subjects could decide in a way that would avoid guilt or regret in subsequent choices. Still, participants could decide on the basis of loss aversion (Kahneman and Tversky, 1984), i.e., choosing the lottery with the lower possible amount of lost money in the case of a loss, which would be a simple and effective strategy to reduce the expected extent of guilt/regret if the chosen lottery does not win^2^. We expected that particularly participants high in TG would be inclined to use such a strategy because – due to their overall increased tendency to experience guilt – they may be more motivated to avoid it. This expectation is based on previous findings that subjects with higher TG activate brain areas that are specific to the experience of guilt (in the orbitofrontal cortex) to a stronger degree than subjects with lower TG scores (Wagner et al., 2011). Although mostly pertinent to interpersonal choices, such a strategy would be expected to be adopted in all choices by individuals high in TG, because they tend to interpret their guilt feelings, more than individuals low in TG, as a general hint at possible threats also to the self (Gangemi et al., 2007). Nevertheless, apart from (and independent of) this expected general effect of TG on loss averse choice tendencies, TG could additionally moderate dynamic, condition-specific effects of the expected (interpersonal) guilt vs. (intrapersonal) regret experiences in trial-by-trial analyses. Here, however, subjects low in TG may be more sensitive. Hence, when directly experiencing negative outcomes of own choices, subjects low in TG, as compared with those high in TG, may be more inclined to use specifically (interpersonal) guilt as a relevant information to be considered in the next choice (in order to avoid repetition of this specific experience). This assumption is based on previous findings that subjects with low prosocial value orientation are most sensitive to guilt-induced enhancement of cooperative behavior (Ketelaar and Au, 2003; De Hooge et al., 2007; Nelissen et al., 2007). Accordingly, applying this directly to the personal inclination to experience guilt, more loss aversion would be expected in subjects scoring low in TG specifically after a negatively evaluated outcome in the interpersonal choice condition (assumed to elicit guilt) than in the intrapersonal choice condition (not assumed to elicit guilt). Furthermore, such an effect should be specifically exerted on the subsequent interpersonal condition, where in contrast to the intrapersonal condition guilt feelings would be imminent in the case of a “bad” choice.

## Materials and Methods

### Subjects

We recruited 23 subjects (10 female) for the experiment, who were paid for participation. All but one were right-handed and had no history of neurological or psychological disorder. Participants’ ages ranged between 19 and 31 years (mean = 23.61 years) and each gave informed written consent. The study was approved by the local ethics committee at the Charité Berlin. One male participant was excluded because he expressed a generally very negative opinion on charity donations, counteracting the basic idea of our study.

### Task and experimental procedure

Before participants were given the instructions to the actual task, they were presented with information about a 4-year old Ukrainian girl called Anastasia, the beneficiary of the earnings in the “other” condition. This information included a short text describing the current situation of the child (with a focus on the nature of her illness and her urgent need of medical treatment) and the organization that collects money for this treatment (“Berlin hilft e.V.”). Subjects were informed that they would subsequently participate in a computer game where money could be won or lost not only for themselves, but also for Anastasia, depending on their choices in the game. It was made clear that there would be a real donation to the charitable organization collecting for Anastasia at the end of the experiment, and as proof, they would ultimately sign the money transfer form not only for themselves but also for Anastasia. As a further proof that Anastasia and the charitable organization “Berlin hilft e.V.” really exists, subjects were shown the internet site of the organization, including photographs of Anastasia. Subsequently, they indicated on rating scales in a short questionnaire their opinion on the usefulness of the organization’s aim to collect donations for Anastasia’s medical treatment, and their momentary impulse to actually donate to Anastasia. These control questions confirmed a generally positive attitude (means ± SEM on rating scales from 0 to 10: 8.41 ± 0.40 for usefulness, 6.41 ± 0.57 for current impulse to donate).

Subjects then read the instruction for the experimental task. As mentioned, the task was based on the well-established paradigm by Coricelli and colleagues (Camille et al., 2004; Coricelli et al., 2005), but we used a visually simplified version as described by Nicolle et al. (2011). Participants were instructed to choose between two “wheel of fortune” lottery gambles on each trial, each featuring a win and a loss outcome with differing probabilities (25, 50, or 75%). The probabilities of their possible financial gain or loss were represented by the relative size of colored sectors of a circle (green for win probabilities and red for loss probabilities). Possible gains and losses in a given trial were indicated by positive numbers on the green part of the respective circle (for the possible gains) and negative numbers on the red part of the respective circle (for the possible losses). These numbers represented Euro cents that could be won or lost in the respective lottery, which could be up to 500 cents per trial. In half of the trials this amount of money was assigned to the subject (“self” condition = intrapersonal), in the other half of the trials it was assigned to a donation to Anastasia (“other” condition = interpersonal). Additionally, as a control condition for choice responsibility (a critical prerequisite for feelings of both guilt and regret), in both the “self” and the “other” condition, subjects could not choose the lottery themselves, but the computer made a random choice, which subjects could only watch passively on the screen (“follow” condition). No or only very low feelings of guilt and regret were expected to occur in these trials due to a lack of felt responsibility. Thus, the design comprised the two within subjects factors “self vs. other” and “choose vs. follow,” with differences between “self” and “other” in the “choose” condition being of primary interest. It should be noted, however, that despite the passivity of the subject in the “follow” trials, these trials were as relevant as the “choose” trials in terms of monetary gain or loss, because their outcomes were still assigned either to the subject or to Anastasia according to the “self”/“other” condition, just as in the “choose” trials.

Altogether, the experiment comprised 128 trials, i.e., 32 per experimental condition, presented in random order. The assignment of specific lottery pairs to experimental conditions was balanced across subjects. An initial capital of 5 Euro was assigned separately to both the subject and to Anastasia, and subjects were told that depending on their decisions, their own as well as Anastasia’s initial capital could increase (to up to more than 50 Euro in the best case) or be lost completely in the course of the experimental game. The task was displayed by Eprime2 software on the screen of a desktop computer located on a table in front of the participant. Before the actual task began, subjects performed a short practice run in order to get familiarized with the basic procedure.

**Figure 1:**
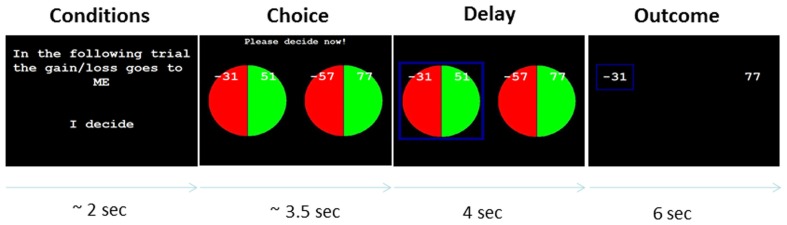
**Exemplary trial**. One of two lotteries was to be chosen in each trial. The outcome of the chosen lottery had actual monetary consequences (gain or loss) for either the participant or Anastasia. In the beginning of each trail, the experimental condition of the trial was announced. There were four possibilities, depending on whether the gain/loss of the chosen lottery would be assigned to the participant or to Anastasia (“self” vs. “other” condition), and whether the participant or the computer would decide which lottery would be played (“choose” vs. “follow” condition). See text, for detailed description of the procedure.

Figure 1 shows an exemplary trial of the task from the start up to the display of the gamble outcome. At the beginning of each trial, subjects were presented with a slide informing them about which of the four possible conditions would follow, i.e., showing information about (i) who they were playing for (“self” or “other” condition) and (ii) whether it would be them or the computer deciding between the two gambles (“choose” or “follow” condition). After subjects indicated by a button press that they had understood this information, the trial itself started by showing the two lotteries, one of which was to be selected.

In “choose” trials, the preferred gamble was selected by the subject by means of pressing either “c” (gamble on the left-hand side) or “v” (gamble on the right-hand side) on the computer’s keyboard. Participants were allowed up to 8 s to make their choice (which was abundant time; mean choice time was 3.49 s). A longer hesitation resulted in a message on the screen reminding them to act faster in the future (which occurred very rarely, on average 1.2 times per subject) before going straight on to the next trial. To ensure that choice times in the “follow” conditions (computer choices) did not differ from the “choose” conditions, the average of the previous three “choose” trials was used as the choice times in the “follow” conditions. Once selected (by either the subject or the computer), the chosen gamble was highlighted on the screen by a blue square, which remained there for 3–5 s (4 s on average). After this delay phase, the outcomes of both gambles (the selected one and the non-selected one) were shown, with the outcome of the selected gamble again being highlighted by a blue square.

After the outcome phase participants were asked to indicate how they felt about the outcome by several subjective ratings. Ratings were provided in a two-stage procedure. First, subjects gave an overall rating on how they felt on a 6-point scale ranging from “very negative” to “very positive.” The scale was presented as six horizontally arranged boxes on the screen, with the two verbal labels as anchors shown on the left (“very negative”) and on the right (“very positive”). Participants were able to move a cursor (initially positioned in the middle) between the six boxes and finally confirmed their choice by pressing the key “m.” Then, in the second step, if the final position of the cursor had been in one of the three boxes symbolizing predominantly negative feelings about the outcome (the focus of interest here), the subject was asked to rate the extent of several specific negative emotions felt, namely guilt, regret^3^, anger/irritation, and disappointment. This was done by means of moving the cursor on a straight line, with no such feeling on the outmost left (verbally anchored as “not at all”) and a very intense feeling being on the outmost right (verbally anchored as “very strong”). All these questions started with the cursor on the outer left (i.e., using no feeling of the respective emotion as the default) rather than in the middle of the scale, to avoid a possible bias of central tendency. The final cursor position had to be confirmed by pressing “m” before the next question appeared. Eleven cursor positions were possible, which were recoded into numbers from 0 to 10. Analogously, if the participant had initially indicated to feel predominantly positively about the outcome (by choosing one of the three rightmost boxes in the first question), he/she was subsequently asked to rate the extent of several specific positive emotions felt, namely joy/happiness, relief, contentment, and pride. In all trials (whether negative or positive), subjects were further asked in the second rating step to indicate to which extent they felt responsible for the outcome (as a control question for the “choose” vs. “follow” manipulation). The five ratings contained in the second rating step were presented in randomized order. Because the negative emotions guilt and regret were in the focus of interest here, analyses of the subjective ratings were limited to the negatively evaluated outcomes.

The two gambles in each presented gamble pair were of equal or nearly equal expected value (maximal difference of 3 cents). In order not to draw attention to this fact, we used uneven values for gains and losses in most of the gambles (avoiding numbers that could be divided by 10), so that an exact calculation of expected values was difficult even after some task practice. (This procedure is the reason why expected values were not always exactly the same between the two gambles, but could deviate by a few cents.) Furthermore, following the procedure of Nicolle et al. (2011), to additionally obscure the fact that expected values in each gamble pair were essentially identical, as well as to enhance feelings of skill in the game, two of the trials in each condition were “catch trials.” This term refers to trials including one gamble with a clearly higher expected value than the other. These “catch trials” were not included in statistical analyses. One catch trial as well as two of the remaining trials in each condition further served as “attention control trials.” In these “attention control trials,” the outcome phase was not followed by subjective emotion ratings but by three questions to determine how well the participant had paid attention to the experimental conditions of the current trial. Specifically, subjects were asked (i) who decided in the current trial (self or computer), (ii) who received the gain/loss in the current trial (self or Anastasia), and (iii) which of the two gambles had the better outcome (the selected one or the non-selected one). Because subjects knew that such control trials would occur repeatedly throughout the experiment, they were forced to keep their attention level high during the entire time. (In fact, analyses on these control trials confirmed this, with only 1.1 mistakes made per person on average.) Since these trials could not be analyzed due to the absence of emotion ratings, there were finally 112 valid trials (28 per experimental condition) for statistical analyses in each subject. Although gambles in these 112 valid trials were mostly played exactly in the way as indicated on the screen (random and independent outcomes) we manipulated the outcome of three trials per condition to make sure that at least some negatively evaluated outcomes expected to be primarily associated with guilt or regret (which was our focus of interest) actually occurred. Specifically, we made sure that the chosen gamble in these trials lost and the non-chosen gamble won. (In turn, to compensate for this bias toward losing, we made sure that the selected gamble always won in the “catch trials” mentioned above).

After the gamble task, which took about 45 min, subjects filled out the TG scale of the Guilt Inventory (Jones et al., 2000), consisting of 20 items rated on 5-point response scales (exemplary items are: “Guilt and remorse have been a part of my life for as long as I can recall,” “I often feel ‘not right’ because of something I have done,” and (with reverse scoring) “Guilt is not a particular problem for me”). Before final debriefing, subjects received their monetary payoff, consisting of a show-up fee of 24 Euro (some of the subjects were psychology students that chose to be reimbursed instead by course credits) and an additional amount based on the outcome of the experimental task. A task-dependent earning was also calculated for Anastasia. As the total expected value across all gamble outcomes was below zero, only few participants actually won anything for themselves or for Anastasia. For ethical reasons, those who lost were informed that they had kept their initial starting capital of five Euros. The same applies to Anastasia. Hence, all participants ended up with additional earnings of at least five Euros for themselves as well as for Anastasia. Participants were informed about their own and Anastasia’s earnings and signed both money transfer forms, and the respective amounts of money were then transferred to the subject and to the charitable organization “Berlin hilft e.V.,” respectively.

## Results

### Comparisons of emotional experiences between “self” (intrapersonal) and “other” (interpersonal) conditions

**Figure 2:**
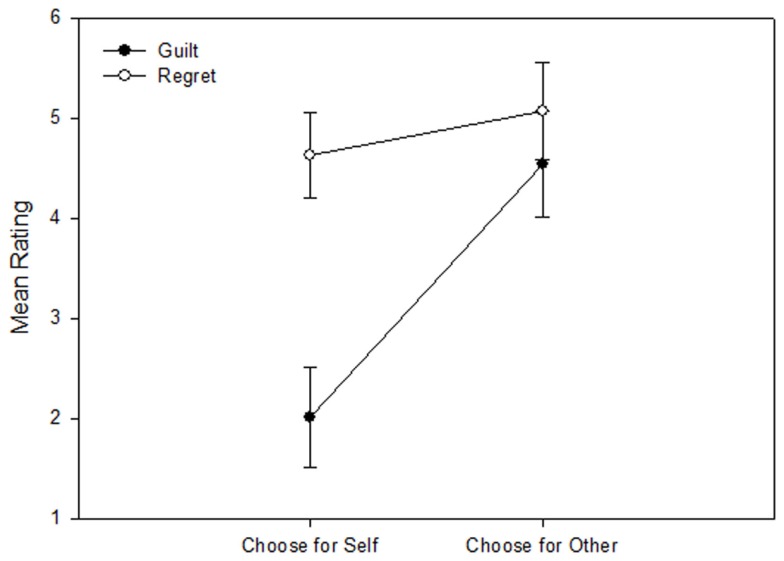
**Ratings of guilt and regret (means ± SEM) after negatively valenced outcomes when subjects had chosen a lottery for themselves (“self” condition = intrapersonal/non-social) or for Anastasia (“other” condition = interpersonal/social)**. Guilt but not regret predominantly occurs in the interpersonal context.

Because the focus of interest was on the negative emotions guilt and regret, the primary analysis comparing the “self” (intrapersonal) vs. “other” (interpersonal) conditions was performed on the negatively evaluated trials (i.e., trials with overall outcome evaluations below the midpoint of the overall negative-to-positive outcome evaluation scale^4^). First, a 2 × 2 ANOVA was performed only on “choose” trials with “guilt vs. regret” and “self vs. other” as within subjects factors, as an analysis directly corresponding to previous psychological studies on intra- vs. interpersonal situation descriptions (Berndsen et al., 2004; Zeelenberg and Breugelmans, 2008). Figure 2 shows the results. Both main effects were significant [“guilt vs. regret,” *F*(1,21) = 25.0, *p* < 0.001; “self vs. other,” *F*(1,21) = 24.7, *p* < 0.001]. Most critically, these main effects were qualified by a significant interaction between the two factors [*F*(1,21) = 22.8, *p* < 0.001]. Subsequent pairwise *t*-test comparisons between “self” and “other” separately for guilt and regret revealed that, as expected, guilt was more strongly experienced in the interpersonal “other” condition than in the intrapersonal “self” condition [*t*(21) = 5.62, *p* < 0.001], while this was not the case for regret [*t*(21) = 1.64, *p* = 0.12], although the trend in the means was in the same direction as for guilt. Pairwise comparisons within conditions further showed that ratings of regret were stronger than ratings of guilt in the “self” condition [*t*(21) = 6.61, *p* < 0.001], but not in the “other” condition [*t*(21) = 1.43, p = 0.17]. The same analysis performed on “follow” control trials (in which guilt and regret ratings were generally very low, as expected; see Table 1) did not reveal any significant effects, confirming specificity of the pattern to conditions of subjectively experienced responsibility. Also, when the factor “choose vs. follow” was directly included as an additional factor in the ANOVA, all respective interactions with this factor were likewise highly significant (*p* < 0.005).

**Table 1:** **Subjective ratings**.

	Choose		Follow	
	Self	Other	Self	Other
Guilt	2.01 (0.50)	4.54 (0.53)	0.42 (0.26)	0.65 (0.29)
Regret	4.63 (0.43)	5.07 (0.49)	1.88 (0.56)	1.69 (0.56)
Anger/irritation	5.74 (0.37)	6.32 (0.41)	5.05 (0.37)	5.61 (0.45)
Disappointment	5.83 (0.37)	6.67 (0.40)	5.47 (0.45)	5.68 (0.42)
Responsibility	5.07 (0.53)	5.54 (0.56)	0.19 (0.11)	0.19 (0.10)

*Values indicate means and SEMs (in brackets) for negatively evaluated outcomes on rating scales ranging from 0 to 10*.

When TG was introduced as an additional between-subjects factor in this ANOVA (two groups of *n* = 11 each, formed by median split), this factor did not moderate the critical guilt/regret × self/other interaction [*F*(1,20) = 0.66, *p* = 0.43, for three-way interaction with TG]. However, TG interacted with the factor “guilt vs. regret” alone, indicating generally enhanced ratings of guilt, but not regret, in high as compared to low TG scorers [*F*(1,20) = 4.22, *p* = 0.05]. Separate analyses performed in high and low TG subjects confirmed a significant guilt/regret × self/other interaction in both groups [strongly enhanced ratings of guilt, but not regret, in the interpersonal as compared to the intrapersonal condition; high TG: *F*(1,10) = 10.02, *p* = 0.01, guilt-other 5.18 ± 0.77 vs. guilt-self 2.78 ± 0.78; regret-other 4.93 ± 0.70 vs. regret-self 4.98 ± 0.64; low TG: *F*(1,10) = 16.43, *p* = 0.002, guilt-other 3.90 ± 0.72 vs. guilt-self 1.23 ± 0.56; regret-other 5.21 ± 0.71 vs. regret-self 4.28 ± 0.59].

For explorative purposes, we also looked for sex differences, using gender instead of TG as a between-subjects factor. In fact, there was a significant three-way interaction with gender, indicating that the critical guilt/regret × self/other interaction was stronger in women than in men [*F*(1,20) = 7.44, *p* < 0.05]. However, because the overall pattern was the same in men as in women and was still significant when calculated separately in men alone [*F*(1,11) = 7.43, *p* = 0.02], we do not consider sexes separately in the interpretation of results.

For the sake of completeness, Table 1 shows means and SEMs also for the other negative emotions anger/irritation and disappointment, as well as responsibility ratings, in all experimental conditions (data not separately shown for the low vs. high TG scorers because of the lack of effects of this factor; all *p*s > 0.12). These data show that guilt and regret, unlike disappointment, and anger/irritation, were generally closely linked to the “choose” condition, where participants felt – in contrast to the “follow” condition – personally responsible for the outcome.

**Figure 3:**
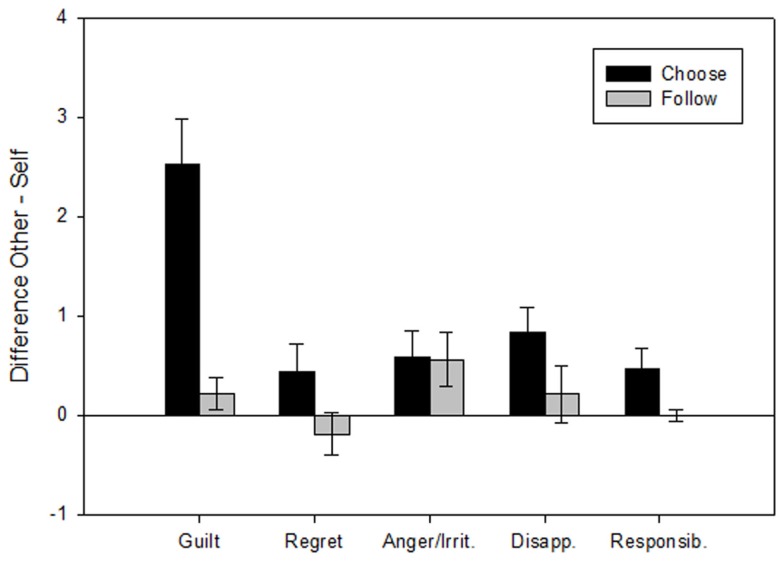
**Rating differences (means ± SEM) between “Other” and “Self” conditions (as an indicator of specificity to a social context) for guilt, regret, anger/irritation, disappointment, and responsibility after negatively evaluated trial outcomes**. In the active “choose” conditions (black bars), guilt showed distinct social specificity, differing from all other emotions (all *p* < 0.001). In passive “follow” conditions (gray bars), no social specificity was observed for any of the ratings.

Because the difference between “other” and “self” was of primary interest here as an indicator of specificity to a social context, we directly calculated in a complementing analysis this difference as a separate dependent variable and compared guilt not only with regret, but also – as an additional control for specificity – for the other negative emotions disappointment and anger/irritation (as well as the control variable of perceived responsibility), and performed the same comparisons not only in the critical “choose” conditions, but also in the no-responsibility control condition of “follow” trials (Figure 3). The figure shows that although all emotions were overall somewhat higher in the “other” than in the “self” condition when subjects had actively chosen the gamble to be played, only guilt showed a distinct specificity in this regard. This was confirmed in a 5 (emotion) × 2 (choose/follow) within subjects ANOVA by an emotion × choose/follow interaction [*F*(4,84) = 11.38, *p* < 0.001] qualifying a choose/follow main effect [*F*(1,21) = 21.23, *p* < 0.001]. In fact, guilt in the “choose” condition differed strongly from all other emotion ratings in this respect (all pairwise comparisons *p* < 0.001). Regarding “follow” control trials (gray columns in Figure 3), there was no consistent pattern, and for none of the emotions did the other-self difference differ significantly from zero.

Despite the differential extent of guilt and regret experiences depending on interpersonal vs. intrapersonal choices, correlation analyses still showed a close relation between responsibility, guilt, and regret even within the “choose” conditions, where responsibility ratings were generally on a high level. In the critical “Other-Choose” condition, the only condition where ratings indicated the experience of both guilt and regret to a substantial degree, both guilt and regret were highly correlated with subjectively perceived responsibility and also correlated with each other (correlation guilt-responsibility, *r* = 0.68; correlation regret-responsibility, *r* = 0.70; correlation guilt-regret, *r* = 0.74; all *p* < 0.001). Even in the “Self-Choose” condition, despite the generally low level of guilt, responsibility correlated with both regret and guilt (correlation regret-responsibility, *r* = 0.77, *p* < 0.001; correlation guilt-responsibility, *r* = 0.44, *p* < 0.05), and guilt and regret ratings also showed a strong direct correlation (*r* = 0.64, *p* < 0.001). (The correlations between guilt and regret even survived when disappointment and anger/irritation were partialed out (*p* < 0.05), but lost significance when responsibility was additionally partialed out.) When TG was correlated with guilt and regret ratings in the Other-Choose condition, the correlation with guilt but not with regret was significant (guilt, *r* = 0.47, *p* < 0.05; regret *r* = 0.17, *p* = 0.46). In the Self-Choose condition, TG likewise correlated with guilt (*r* = 0.49, *p* < 0.05) but also with regret (*r* = 0.42, *p* = 0.05).

### Trait guilt and loss aversion

To test the hypothesis that higher TG is associated with stronger loss aversion, we first compared low and high TG scorers with respect to the overall number of trials in the “choose” condition in which they preferred the option with lower possible loss over the option with higher possible loss, using a *t*-test for independent samples. This analysis revealed a significant difference, with a higher percentage of loss averse choices for high as compared to low TG subjects (23.2 ± 2.8% vs. 15.3 ± 2.1%, *t*(20) = 2.25, *p* < 0.05).

To test the additional possibility of a more dynamic and more specific influence of TG on choice behavior, we first computed four different variables for each subject: (1) the percentage of negatively evaluated other-choose trials followed by a loss averse choice in the next other-choose trial (“Other–Other” condition), (2) the percentage of negatively evaluated other-choose trials followed by a loss averse choice in the next self-choose trial (“Other-Self” condition), (3) the percentage of negatively evaluated self-choose trials followed by a loss averse choice in the next self-choose trial (“Self–Self” condition), and (4) the percentage of negatively evaluated self-choose trials followed by a loss averse choice in the next other-choose trial (“Self-Other” condition). The first two of these variables represent the conditions of choices after experiencing (interpersonal) guilt, while the latter two variables represent the conditions of choices after experiencing (intrapersonal) regret. Any specific effects of these emotions would be reflected in selectively enhanced values in congruent choice conditions corresponding to these emotions, i.e., the “Other–Other” condition for specific effects of guilt and the “Self–Self” condition for specific effects of intrapersonal regret. Furthermore, no such effects should occur when a previous choice was not negatively but positively evaluated due to the absence of guilt or regret feelings. For control purposes, we therefore calculated the same four variables also for the choices after positively evaluated outcomes. Thus, the percentage of loss averse choices in these different conditions was subjected to an ANOVA, with the three within subjects factors “self vs. other” in current trials, “self vs. other” in the next “choose” trial, and “negative” vs. “positive” emotional evaluation of the current outcome, and the additional between-subjects factor low vs. high TG.

This ANOVA showed that, apart from a main effect of high vs. low TG [*F*(1,20) = 5.05, *p* < 0.05, confirming the above mentioned overall *t*-test comparison between the two groups], emotion-specific effects indeed occurred depending on TG. Specifically, a main effect of “self vs. other” in the next choice was qualified not only by an interaction with valence (“negative” vs. “positive”) but also with “self vs. other” in the current choice in combination with TG [all *F*(1,20) > 4.5, *p* < 0.05]. To break down these differential effects depending on TG, we analyzed the emotion-specific patterns separately in the groups of high vs. low TG subjects. Within the high TG group, only the main effect of “self vs. other” in the next choice was statistically significant, indicating overall higher loss aversion in “Self” than in “Other” conditions [*F*(1,10) = 6.65, *p* < 0.05]. In contrast, within the low TG group a more complex, emotion-specific pattern was observed, in which a main effect of valence was qualified by an interaction with “self vs. other” in the next choice, and by an interaction with “self vs. other” in the next choice in combination with “self vs. other” in the current choice [all *F*(1,10) > 8.3, *p* < 0.05]. Inspection of the mean values in the different conditions (Table 2) shows that this interaction reflects an effect specifically related to the condition of guilt experience (negative Other condition) in this group of subjects. In fact, subjects in this group, just as those in the high TG group, likewise showed an overall tendency toward higher loss aversion in decisions for oneself in all conditions, while only after experiencing guilt this pattern was reversed (i.e., higher loss aversion in decisions for Anastasia). Directly correlating TG with loss averse choice behavior confirmed the ANOVA results. TG was positively correlated [*r* = 0.37] with loss aversion overall (reflecting the main effect), but negatively correlated specifically with the difference between loss averse interpersonal choices after guilt experience (i.e., negative Other–Other condition) and respective control conditions on which the complex interactive effect is based (difference to negative Other–Self, *r* = −0.65; difference to positive Other–Other, *r* = −0.41; each *p* < 0.05).

**Table 2:** **Percentages of loss averse choices**.

	Current choice	Self		Other	
	Next choice	Self	Other	Self	Other
**High trait** **guilt**	Negative evaluation	23.3 (3.8)	18.3 (5.5)	32.1^a^ (4.7)	21.2^a^ (4.5)
	Positive evaluation	24.3 (4.3)	17.4 (3.6)	31.8^b^ (6.5)	17.5^b^ (1.3)
**Low trait** **guilt**	Negative evaluation	18.3 (2.1)	15.6^c^ (3.7)	12.8^d^ (3.0)	23.2^c,d,e^ (3.8)
	Positive evaluation	14.3 (2.3)	11.1 (3.5)	16.3 (4.3)	10.8^e^ (2.7)

*Values indicate means and SEMs (in brackets) for percentages of subjects’ loss averse next choices when they evaluate the outcome of their choice in the current trial as emotionally negative vs. emotionally positive, depicted separately for the four possible combinations of choice conditions (“Self” followed by “Self”/“Self” followed by “Other”/“Other” followed by “Self”/“Other” followed by “Other”) and for participants scoring high vs. low on Trait Guilt (*n* = 11 per group). Current “Self” choice with negative emotional evaluation is associated with (intrapersonal) regret, current “Other” choice with negative emotional evaluation is associated with (interpersonal) guilt. High Trait Guilt scorers are generally more loss averse, but unlike low scorers do not change their choice behavior in response to specific emotional experiences in the current choice trial. Values with identical superscript differ significantly from each other in pairwise comparisons between experimental conditions at *p* < 0.05*.

A control analysis using risk-avoiding choice behavior as the dependent variable (choosing the lottery with the lower difference between the possible gain and the possible loss) did not reveal any statistical significance (all ANOVA effects *p* > 0.12), showing specificity of the observed effects to the simpler choice strategy of loss aversion, in which possible gains are not considered.

Although women and men did not differ significantly in TG in our sample [women 54.16 ± 3.37, men 50.56 ± 3.61, *t*(20) = 0.72, *p* = 0.48], we also performed a control analysis on loss averse choices using gender instead of TG as between-subjects factor. None of the effects including gender reached significance (all *p* > 0.31), excluding the possibility that the differential effects observed for low vs. high TG subjects would simply reflect gender differences.

## Discussion

Extending a well-established experimental procedure of intrapersonal regret (Camille et al., 2004; Coricelli et al., 2005) by adding an interpersonal (social) dimension, we present here an experimental procedure which allows a differential induction of feelings of guilt and regret. Specifically, in accordance with previous psychological findings based on descriptions of scenarios or personal past events, guilt was induced to a stronger degree when subjects felt responsible for interpersonal harm than when they felt responsible for intrapersonal harm. This was not the case for regret, nor for disappointment, or anger/irritation, although the means in all these emotions tended into the same direction as for guilt. Not only the clear pattern of results that confirmed the primary hypothesis on the differences between guilt and regret is remarkable, but also the absolute intensity of these feelings elicited in this very simple choice paradigm (means of about five on a scale ranging from 0 to 10 in the conditions of interest). As to regret, we had not formulated a specific hypothesis, because previous results were inconsistent with regard to the intrapersonal vs. interpersonal nature of regret (Berndsen et al., 2004; Zeelenberg and Breugelmans, 2008). In the present study, we found no substantial difference between regret ratings between intrapersonal and interpersonal choice conditions, consistent with the findings from Zeelenberg and Breugelmans (2008).

Despite the clearly different pattern of means for guilt and regret between conditions, our results obtained from correlation analyses also confirm the specific commonalities of these two emotions. In contrast to other negative emotions, guilt and regret were closely associated with subjectively experienced responsibility. Even within “choose” conditions (associated with high responsibility) both guilt and regret correlated substantially with subjective responsibility, and they were also highly correlated with each other. Particularly in interpersonal choice conditions, where both guilt and regret ratings had similarly high absolute values, this raises the interesting question of what conceptually distinguishes guilt from interpersonal regret. Obviously, while there can be regret without substantial guilt (as shown in the intrapersonal condition), it may be impossible to experience guilt without regret in interpersonal conditions, and the high correlation between the two emotions suggests that they do not only co-occur in these contexts, but indeed strongly overlap conceptually. In other words, guilt and interpersonal regret may describe essentially the same core emotion. Alternatively, there may still be differences in the sense that, contrary to interpersonal regret, guilt is not only linked to a social context but also to moral evaluations in a certain situation. A closely related question would be whether regret experienced in an intrapersonal context is qualitatively the same as regret experienced in an interpersonal context. If not, it would be useful to use different names for them, or to always add the respective adjective describing the particular context. The present study was not designed to answer these questions, which should be investigated more directly in future studies. In the following, we will refer to regret only as intrapersonal regret, not only because this is the traditional use of the term in regret research (Mellers et al., 1999; Camille et al., 2004; Coricelli et al., 2005), but also due to its obvious close link to guilt in the interpersonal domain.

It is noteworthy that subjects indicated strongly enhanced feelings of responsibility (and as a consequence also of regret and guilt) after own lottery choices, compared to those made by the computer, even though the final outcome was still a matter of chance. (Subjects did not have to choose the outcome directly, but only the lottery to be played.) This underlines the role of subjective responsibility, as opposed to objective responsibility, as critical factor underlying feelings of guilt and regret. Although objective and subjective responsibility would normally coincide, the latter appears to be the primary determinant if they do not. For example, people typically feel more regret when a negative outcome is a result of their action rather than of their inaction (Kahneman and Tversky, 1982), which can be explained by reduced sense of responsibility for inaction. Conversely, subjectively perceived responsibility may be experienced in certain cases of “survivor guilt” in people who are the only survivor of a traffic accident, even though they were not involved in any way in the circumstances leading to the accident. Thus, although our simplified paradigm does not simulate a prototype of an everyday situation of guilt and regret, it still captures subjective responsibility as a central factor.

A specific advantage of the present paradigm, consisting of a series of personal choices, is that it not only allows the differential induction of guilt and regret, but also examining how avoiding these emotions can affect choice behavior. Because such effects are likely to be influenced by personality differences pertinent to these emotions (De Hooge et al., 2007; Gangemi et al., 2007; Nelissen et al., 2007), we focused here on guilt, for which, in contrast to regret, established procedures to assess stable individual differences exist (see Robins et al., 2007, for an overview). In the present paradigm, subjects had few opportunities to employ complex choice strategies in order to avoid guilt or regret, and there was no objectively better choice option because in each trial both lotteries had comparable expected values. Given these constraints of the task, we assumed that subjects motivated to avoid guilt or regret would apply the simple strategy of loss aversion by choosing the lottery in which less money was lost in the case of a loss. We found two interesting results in this regard. First, subjects high in TG were generally more loss averse than those low in TG. Second, however, only subjects *low* in Trait displayed enhanced loss aversion in the next emotion-congruent trial (next interpersonal choice) after an antecedent experience of guilt. Although seemingly contradictory at first glance, this pattern makes sense. Subjects high in TG are probably most strongly motivated to avoid guilt. As confirmed by our data, these subjects did indeed exhibit overall higher guilt ratings after choices with negative outcomes. Because this was likewise the case for interpersonal as well as for intrapersonal choices, it makes sense that these subjects generalize their motivation to avoid guilt to all decisions. From this perspective, loss aversion *per se* in risky choices may represent a stable trait-like factor, which is linked to high TG. In contrast, low TG scorers, who do not feel guilt as frequently, may be able to use their guilt and regret feelings more readily as helpful information that they can use to adapt future behavior specifically under similar circumstances.

Thus, our results confirm previous studies also pointing to personality-dependent effects of guilt-associated behavior. In a study from Ketelaar and Au, 2003, Study 1, effects of experimentally induced guilt led to increased cooperation in a repeated prisoner’s dilemma game only in subjects who initially played uncooperatively (a behavior likely to be associated with low TG), while participants who played highly cooperatively from the beginning, showed no effect of guilt induction. Such effects may be explained by a ceiling effect, but this explanation would not be convincing because subjects were clearly below ceiling at least in some of the conditions, and even more so in the present study, where the overall percentage of loss averse choices was on average below 25% even in high TG subjects^5^. The findings by Ketelaar and Au (2003) were further supported by subsequent studies demonstrating that only proself-oriented, but not prosocial subjects were particularly sensitive to effects of guilt induction procedures on subsequent cooperation in a one-shot social dilemma game (De Hooge et al., 2007; Nelissen et al., 2007).

Consistent with these previous results, and in line with the original interpretation from Ketelaar and Au (2003), we would therefore take our findings as a support for a functional view of the “affect-as-information” model (Schwarz and Clore, 1983), where using the own affective state (in this case: feelings of guilt) as an information for future behavior is most effective in individuals who are not too strongly accustomed to experiencing these emotions. If experiencing certain emotions becomes a habit or trait (as in the case of guilt in high TG scorers), these emotions may become less informative, and a more general pattern of choice behavior (a general loss aversion here) may emerge. This more general effect, found to be related to high TG in our study, is in line with a previous study by Gangemi et al. (2007) who showed that individuals high in TG more than those low in TG use their guilt feelings as information about possible threats when anticipating types of negative events in which oneself feels responsible and which would potentially lead to damage not only to others, but also to oneself.

Our analysis focused on loss aversion, because we assumed that subjects’ differential motivation to avoid guilt or regret would most likely be reflected in this behavioral strategy as the easiest possible strategy that subjects could apply within the constraints of our experimental paradigm. Consistent with this assumption, we did not observe differences between experimental conditions when we performed the same analysis on risk-focused rather than simply loss-focused behavior, i.e., a strategy that compares the two lotteries not only with regard to possible losses but with regard to the difference between gains and losses within each lottery. Such risk-focused behavior is more cognitively demanding in our task because it requires taking four rather than only two numbers into account as a basis for the decision. However, these results do not imply that loss averse choice behavior would generally be the preferred strategy that people apply. Depending on specific circumstances of a task (e.g., number of trials, time limits for decisions) risk-focused strategies can likewise be used. We would expect this particularly if such a strategy is less cognitively demanding than in our task. This is in fact the case in the more prototypical studies related to risk aversion vs. risk seeking, where subjects choose between a gamble and a safe option, so that the risk differences between the two alternatives are obvious. Actually, behavioral effects of anticipated regret and guilt have previously been shown in such tasks (e.g., Zeelenberg et al., 1996; Mancini and Gangemi, 2003). The influence of task-dependent cognitive load is certainly a relevant aspect that would deserve closer examination in future studies.

In sum, the results on the effects of emotional experiences on choice strategies in our paradigm overall demonstrate basically two ways in which guilt and regret could exert self-regulating effects via an influence on loss averse choice behavior. One is a general one linked to high TG, i.e., an individual tendency to experience guilt (but most likely also to some degree to the tendency to experience regret, because the TG scale does not unambiguously differentiate between the two emotions), which leads to generally enhanced loss aversion in decisions. This way, these individuals can generally minimize the occurrence of both guilt and regret. The second way is a situation-specific effect where interpersonal guilt and intrapersonal regret experiences lead to behavioral changes only in subsequent congruent experimental conditions, i.e., when interpersonal guilt and intrapersonal regret can be avoided, respectively. However, this second effect is not independent of the first one, because it is only found in individuals with low TG in relation to guilt avoidance. It can therefore be regarded as an alternative strategy for those subjects who do not adopt the general strategy, as the high TG scorers do.

Both strategies can be interpreted within the framework of “indirect causation theory” (Baumeister et al., 2007), which proposes that consciously experiencing emotions enables people to learn from their experiences. Specific evidence for this view with regard to guilt comes from self-reports of people who typically indicate that they have learned something from personal events in which they had experienced guilt feelings (Baumeister et al., 2007; Stillman and Baumeister, 2010). Our data suggest that one behavioral indicator of this learning process is expressed in loss aversion (consistent with an attempt to avoid feelings of guilt and regret), but what exactly has been learned in this regard appears to differ between individuals high vs. low in TG as a result of different learning histories associated with guilt experiences. Whereas the former group, being prone to guilt in all choice situations (as confirmed by our subjective rating data), apparently learned a general lesson to avoid guilt (but also all other associated negative emotions, including regret) in decision situations, the latter group seems to have learned more specifically to behave in a way that avoids the repetition of a specific guilt experience having occurred shortly before (cf. “feeling-is-for-doing” approach by Zeelenberg et al., 2008). Put differently, the first group may have adapted their behavior more generally on the basis of their overall enhanced guilt experiences, regardless of their occurrence in a specific situational context, while the other group rather learned to use these emotions to change behavior acutely within a situational context.

Regarding differences between guilt and regret, at least the second strategy appears to be clearly emotion-specific, being confined to conditions of interpersonal choice, and hence experiences of guilt. However, two possible caveats are to be considered here. First, our data show a generally higher loss aversion in “self” choices than in “other” choices. This might indicate that subjects’ motivation to avoid intrapersonal regret was overall higher than the motivation to avoid guilt, and could therefore less easily be further enhanced by additional motivational effects of personality factors. Second, and more importantly, we focused our analysis here on TG as a moderating personality factor. It is likely that analogous results could be found for regret avoiding decisions in relation to personality factors specifically related to regret proneness. It would be useful to include such a personality factor in future studies in order to strengthen the interpretation of emotion specificity of behavioral effects of guilt vs. regret. Most desirable for this purpose would be the development of an instrument that specifically aims at distinguishing guilt- and regret-related trait factors. To our knowledge, such an instrument is still missing, while much work has been devoted to create differential measures for guilt and shame proneness (Tangney, 1990; Kugler and Jones, 1992; Robins et al., 2007; Rüsch et al., 2007; Tangney et al., 2007).

In conclusion, we developed a new experimental decision-making paradigm that allows a differential induction of guilt and regret online (despite the close relatedness of these two emotions), as well as an analysis of their effects on regulation of subsequent choice behavior. The results show that TG is a critical factor that moderates the role of guilt vs. regret avoidance as critical regulators of choice behavior by way of loss averse strategies. Although definite conclusions regarding the differential self-regulating functions of guilt vs. regret would be premature at this stage, the data suggest that feelings of guilt are mostly informative in acute, short-term decisions for those people who do not experience them often. However, if experienced more regularly and intensely, guilt may exert behavioral and emotion regulating effects that go beyond the short-term anticipation of its occurrence, resulting in a more generalized strategy to avoid guilt with its associated negative emotions (including regret). If confirmed, it would be interesting to investigate how such processes can contribute to certain clinical conditions, such as obsessive compulsive disorder, borderline personality disorder, and major depression, which are associated with enhanced guilt propensity (Mancini and Gangemi, 2004; Rüsch et al., 2007; Kim et al., 2011). It is conceivable that in certain extreme cases, where guilt is increasingly experienced even without any reasonable justification, such generalized effects of guilt on choice behavior and decision-making may become more and more maladaptive and could in this way eventually lead to “pathological guilt” as observed in such disorders (Shapiro and Stewart, 2011). Because our results point to a critical role of stable individual differences, it would be useful to develop differential trait questionnaires techniques that can better distinguish between the inclinations to experience guilt vs. regret than it is possible at present. Such an improved distinction would be relevant not only theoretically, but may ultimately also be useful to understand how exactly guilt- vs. regret-related regulation mechanisms contribute to the etiology of psychiatric disorders in which these emotions play a critical role.

## Conflict of Interest Statement

The authors declare that the research was conducted in the absence of any commercial or financial relationships that could be construed as a potential conflict of interest.

## Footnotes

^1^We did not formulate an opposite hypothesis for regret, i.e stronger regret experiences in the intrapersonal than in the interpersonal context. Based on the previous findings from Berndsen et al. (2004) and Zeelenberg and Breugelmans (2008), it seems to depend on specific circumstances of a situation whether regret per se would differ between intra- and interpersonal conditions. Accordingly, our focus is more on guilt than on regret here, and on the *difference* between guilt and regret. Therefore, whatever the effect on regret per se would be, we would in any case expect an interaction in that the propensity to be experienced more strongly in interpersonal as compared to intrapersonal contexts would be clearly more pronounced for guilt than for regret.

^2^An alternative possible strategy would be to compare both gambles in terms of risk as defined by the difference between the possible gain and the possible loss within each gamble. However, we expected that within the constraints of our paradigm, which includes repeated testing with limited decision time, subjects would rather rely on the less cognitively demanding strategy of loss avoidance, in which only two numbers have to be compared.

^3^There are two possible German translations for “regret”, i.e. “bedauern” and “bereuen”. In our experiment, we used the latter one, which more than the former one implies personal responsibility. We used the verb form “bereuen” because the corresponding noun “Reue” has a religious connotation (similar to repentance) and would normally not be used with respect to simple decisions made in everyday life.

^4^On average, each experimental condition was represented by 14.2 trials, without differences in the amount of trials between conditions (*p* > 0.20). The broad majority of these trials (88%) was associated with both an absolute financial loss (i.e., the chosen lottery lost) and a relative financial loss (i.e., the non-chosen lottery won, or it lost less than the chosen lottery). On average, the absolute monetary loss was 2.17 ± 0.05 Euro and the relative monetary loss was 2.15 ± 0.15 Euro in these trials, without any significant differences between experimental conditions (*p* > 0.25 for all main effects and interactions). Thus, differences in experienced emotions between conditions cannot be explained simply by differences in objective outcomes.

^5^The generally low level of loss-averse choices may be explained by the fact that subjects knew that overall not more than the initial capital could be lost. Hence there was no overall risk to really lose money in the experiment, which may bias subjects toward optimistic choices.
